# Effect of lidocaine spray on relieving non-coring needle puncture-related pain in patients with totally implantable venous access port: a randomized controlled trial

**DOI:** 10.1007/s00520-023-07910-4

**Published:** 2023-07-08

**Authors:** Ying Zhu, Sihua Niu, Yejun Zhang, Huiyan Zhang, Jian Chang, Liqin Ye

**Affiliations:** Department of Nursing, Shanghai General Hospital, Shanghai Jiao Tong University School of Nursing, No. 650, Xinsongjiang Road, Songjiang District, Shanghai, China

**Keywords:** Lidocaine spray, Totally implantable venous access port, Non-coring needle, Pain, Nursing

## Abstract

**Purpose:**

Patients with the placement of a totally implantable venous access port (TIVAP) commonly suffer from pain caused by inserting a non-coring needle. At present, lidocaine cream and cold spray are extensively used for pain management, but they are complex to manage in busy medical environments and developing countries. The lidocaine spray combines the analgesic effect of lidocaine cream and the rapid onset of cold spray, which can effectively alleviate the pain related to non-coring needle puncture in patients with TIVAP. This randomized-controlled trial aimed to explore the effectiveness, acceptability, and safety of lidocaine spray in relieving the pain of non-coring needle puncture in patients with TIVAP.

**Methods:**

A total of 84 patients who were hospitalized in the oncology department of a Grade III Level-A hospital in Shanghai from January 2023 to March 2023 and were implanted with TIVAP and required non-coring needle puncture were selected as the study subjects. The recruited patients were randomly assigned to the intervention group and the control group (*n*=42). Before routine maintenance, the intervention group received lidocaine spray 5 min before disinfection, while the control group received water spray 5 min before disinfection. The main clinical outcome was pain, and the degree of puncture pain in both groups was evaluated using the visual analogue scale.

**Results:**

There were no significant differences between the two groups in age, gender, educational level, body mass index, port implantation time, and disease diagnosis (*P*>0.05). The pain score in the intervention and control groups was 15.12±6.61mm and 36.50±18.79mm, respectively (*P*<0.001). There were 2 (4.8%) patients with moderate pain in the intervention group and 18 (42.9%) patients with moderate pain in the control group (*P*<0.001). In the control group, 3 (7.1%) patients reported severe pain. The median comfortability score for the two groups of patients was 10, but there was a difference between the two groups (*P*<0.05) because the intervention group tilted to the right. The successful puncture rate of the first time puncture had no difference between the two groups, both being 100%. Moreover, 33 patients (78.6%) in the intervention group and 12 patients (28.6%) in the control group reported that they would choose the same spray for intervention in the future (*P*<0.001). During the 1 week of follow-up, 1 patient in the intervention group developed skin itching (*P*>0.05).

**Conclusions:**

The local use of lidocaine spray in patients with TIVAP is effective, acceptable, and safe to alleviate the pain caused by non-coring needle puncture.

**Trial registration:**

Chinese Clinical Trial Registry (registration number: ChiCTR2300072976)

## Introduction

The totally implantable venous access port (TIVAP) is a subcutaneous infusion system that can be retained in the human body for a long time [1]. By virtue of its advantages such as less visibility, facilitating daily activities, causing less catheter-associated bacteremia and thrombosis, enabling high quality of life, and bringing high satisfaction [2–6], TIVAP has been widely used in cancer patients in recent years [7]. The infusion or maintenance period after port placement requires puncture with a non-coring needle, but patients often complain of significant pain during the puncture as the structure of the non-coring needle slows down its speed through the skin [8]. It is reported that the incidence of mild and moderate to severe pain caused by TIVAP needle insertion is 67.5% and 34.9%, respectively [9], and the pain score can reach 3.91 ± 1.35 [10]. The pain caused by needle puncture is related to the fear and suffering of patients [11]. Therefore, reducing the pain during non-coring needle puncture can alleviate the psychological burden of patients and improve treatment compliance.

The pain management of non-coring needle puncture is mainly divided into non-pharmacology and pharmacology, such as the use of virtual reality, Vapocoolant spray, cryotherapy, cutaneous stimulation therapy, lidocaine cream, and lavender essential oil [8, 9, 12, 13]. The Shanghai Expert Consensus on Totally Implantable Venous Access Port 2019 [14] proposes that the pain relief needs of patients should be evaluated before needle insertion, and local anesthetics such as Vapocoolant spray and lidocaine can be considered. Although the Vapocoolant spray has a fast onset time, its surface anesthesia time is relatively short and the operator needs to inject the needle quickly within 30 s [8, 15], which leads to high competency requirements and requires assistance from other operators, and moreover, Vapocoolant spray is less effective than lidocaine for analgesia in intravenous cannulation [16]. Currently, the commonly used lidocaine cream in the clinic needs to be applied 30 to 60 min before operation to ensure the analgesic effect [17], which reduces the work efficiency of nurses and is not suitable for patients in urgent need of infusion.

The main component of lidocaine spray is lidocaine, which achieves the surface anesthetic effect by accumulating at the cortical pain receptors and nerve endings. Local anesthesia can be produced 1–2 min after spraying, and the duration is 15–20 min. Compared with lidocaine cream and Vapocoolant spray, lidocaine spray has the advantages of quick onset, long duration of anesthesia, and convenient use. Some researchers have used lidocaine spray to relieve pain related to venipuncture, radial artery puncture [18, 19], insertion of intrauterine device [20], and thoracic tube removal [21], but some studies have also shown that lidocaine spray cannot effectively relieve local pain caused by intravenous intubation [22]. At present, the pain control of lidocaine spray in the non-coring needle puncture into the TIVAP remains unclear. Herein, we evaluated the effectiveness, acceptability, and safety of the local use of lidocaine spray to alleviate needle insertion pain in adult patients implanted with TIVAP by comparing it to the analgesic effect of the control group (water spray).

## Methods

### Study design and participants

In this parallel randomized controlled trial, 84 patients who were hospitalized in the oncology department of a Grade III Level-A hospital in Shanghai from January to March 2023 were selected as the study subjects. All these patients were implanted with a chest wall TIVAP and required non-coring needle puncture for infusion. After admission, these patients were randomly assigned to the intervention group (42 cases) and the control group (42 cases). Inclusion criteria were as follows: ① age ≥ 18 years old; ② no history of mental illness, normal cognition, and clear language expression; ③ normal pain response; ④ intact skin at the injection port, without damage, redness, or infection. Exclusion criteria were the following: ① long-term use of analgesic drugs; ② allergic to lidocaine. This study was approved by Human Trial Ethics Review Committee of Shanghai General Hospital, and all subjects provided informed consent and voluntarily participated in the study.

### Random grouping

In Excel, a column of numbers 1–84 was established as column A. After numbering, column B of 84 corresponding random numbers was generated using a random function. Column A and column B were all selected and sorted in ascending order of column B, so that 84 numbers in column A were randomly arranged, with the first 42 random numbers assigned to the control group and the last 42 random numbers assigned to the intervention group. Then, the 84 randomly numbered cards were sequentially placed into 84 envelopes and the envelopes were ordered. After the patient agreed to participate in this study, the operator opened the envelopes sequentially according to the envelope number and obtained the random number and corresponding grouping. The patient did not know the grouping situation.

### Sample size calculation

In this parallel randomized controlled trial, the intervention group was subjected to lidocaine spray treatment, while the control group was a blank control, and the pain score was the primary outcome indicator. According to previous literature [10], the pain caused by non-coring needle puncture at the TIVAP was 3.91 ± 1.35 in the control group and 1.57 ± 0.68 in the lidocaine cream intervention group. Setting α=0.05 (bilateral) and β=0.10, the sample size in the intervention group and the control group was calculated as N1=N2=5 using the PASS11 software. Assuming that the dropout rate of the study subjects was 10%, the sample size N1=N2=5 ÷ 0.9=6 was required. Finally, 84 patients were included against the actual situation.

### Procedures

#### Materials

The lidocaine spray (N-(2,6-xylenyl)-2-(diethylamino) acetamide) used in the intervention group was produced by Shanghai Xinyi Pharmaceutical Co., Ltd., a solution type, 8 g per bottle, containing 450 mg lidocaine, 4.5 mg lidocaine per spout, and 100 spouts per bottle. The spray had been approved by the China Food and Drug Administration for local anesthesia for minor operations in the mouth, nose, and throat, with a bottle of lidocaine spray costing 66 yuan RMB.

The spray used in the control group was mineral water spray, which was installed in a hand-held pressurized spray tank similar in size to lidocaine spray. The mist provided a cool and comfortable feeling to the patients.

#### Treatment methods for the control group

Before routine skin disinfection, the water spray was applied twice at about 10 cm away from the puncture point, with an interval of 1–2 min and 3 spouts each time, and skin disinfection was performed within 1–2 min after spraying. Afterward, the standard puncture was performed according to the conventional unified and standardized operation procures: strictly disinfect; wear sterile gloves; fix the injection port with the thumb, index finger, and middle finger of the left hand; merge the two wings of the butterfly wing needle with the thumb and index finger of the right hand and hold it steady; then, insert the needle vertically, slowly, and gently; when there was a feeling of empty space, slowly insert the needle downward for about 0.5 cm, withdraw the needle, accompanied by blood returning, which confirmed that the needle reached the reservoir; immediately flush the TIVAP with 20 mL physiological saline in a pulsed manner, clamp the extension tube, separate the syringe, connect the needle-free airtight connector, and then connect the infusion device for infusion.

#### Intervention methods for the intervention group

Before routine skin disinfection, the lidocaine spray was applied twice at about 10 cm away from the puncture point, with an interval of 1–2 min, 3 spouts each time, 4.5 mg per spout, and a total amount of 27 mg. After spraying, skin disinfection was performed in 1–2 min, and then, the standard puncture was carried out according to the conventional unified and standardized procedures. The puncture method was the same as that of the control group.

The two groups of patients received puncture with a 20 G non-coring needle (B. Braun Melsungen AG, Germany) in the prone position. The operating nurses were all specialized intravenous therapy nurses with more than 5 years of work experience.

### Outcome indicators

#### Pain

The primary outcome measure was the patient’s pain evaluated using the visual analogue scale (VAS) of 0–100 mm from no pain to severe pain. Patients were asked to mark with a vertical line on the 100 mm horizontal scale amount of pain they experienced during the puncture: 10–30 mm mild pain, 40–60 mm moderate pain, and 70–100 mm severe pain. When using, the graduated side of the pain scale was facing away from the patient, and the patient was asked to mark the corresponding position on the scale that represented their own pain degree. The nurse gave a score based on their marked position. Compared to the verbal description scale, the VAS has proven to be more sensitive to small differences in pain intensity and pain discomfort, with higher test repeatability [23]. Notes for use were as follows: ① before use, it is necessary to provide a detailed explanation to the patient, so that the patient can understand the concept of this method and the relationship between the pain measured by this method and real pain, and then, the patient is asked to mark the corresponding position of their pain on the straight line. ② A vernier scale ranging from 0 to 100 mm on the back and a 0–100-mm visual analogue scale on the front can be used. If the patient moves the scale, the nurse can immediately see the specific number on the back of the scale, accurate to millimeters.

#### Acceptability

The patient was inquired whether he was willing to continue to choose this spray when conducting a non-coring needle puncture at the TIVAP in the future, and the answer options were yes, not sure, and no.

#### Comfortability

The numerical rating scale (NRS, 0–10) was used for assessing the self-reported comfortability of patients after maintenance: with 0 representing very uncomfortable and 10 representing very comfortable. Moreover, the numerical rating scale has also been widely used in patient comfortability assessment [24].

#### Success puncture rate of the first time puncture

The proportion of patients with successful puncture at the first time was calculated.

#### Adverse reactions

After the puncture, a follow-up of 1 week was conducted to observe the occurrence of adverse reactions such as local skin redness, whitening, itching, and allergy.

### Data collection methods

All the above indicators were designed as paper questionnaires. With the exception of adverse skin reactions observed and recorded by the quality controller, other indicators were reported by the patients themselves. The quality controller used unified guidelines to explain the requirements for completing the questionnaire and explain questions. The questionnaires were retrieved and checked for omissions or obvious logical errors.

### Data analysis

The SPSS 21.0 software was used for statistical analysis. The measurement data were tested for normal distribution using Kolmogorov-Smirnov test, and those conforming to normal distribution were expressed as mean ± standard deviation ($\overline{x}$± s) and compared using the *t*-test; those not conforming to normal distribution were expressed as median and quartile range [*M* (QR)] and compared using the Mann-Whitney U test. The count data were expressed as number of cases and composition ratio [*n* (%)] and compared using the *χ*^2^ test. A value of *P* < 0.05 was indicative of statistical significance.

## Results

### Comparison of general information between the two groups of patients

**Fig. 1:**
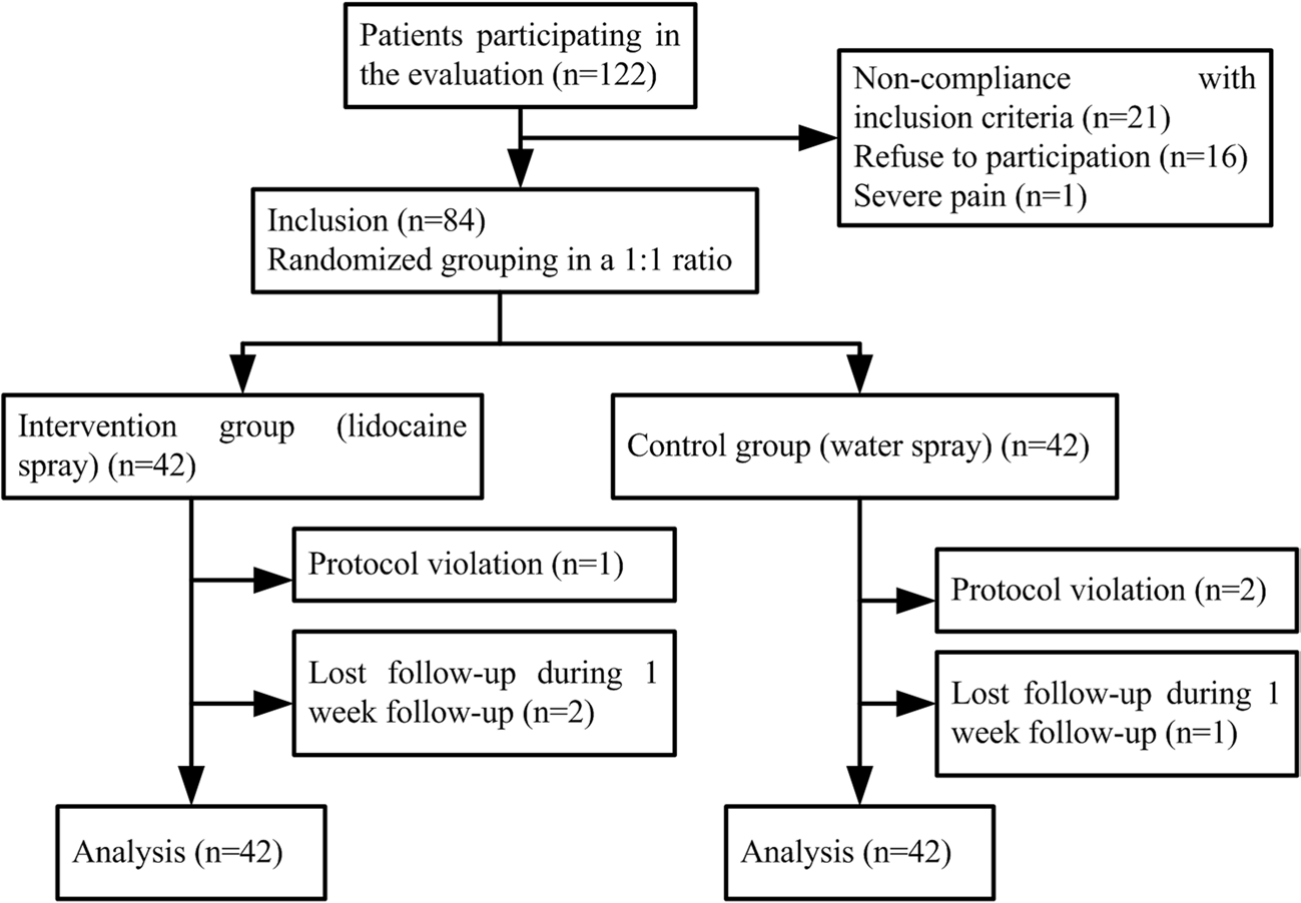
Recruitment and flow of patients

The 84 patients recruited were randomly allocated to the intervention group (*n*=42) and the control group (*n*=42), as shown in Fig. 1. The TIVAP for all patients is placed on the right chest wall. Among the patients receiving non-coring needle puncture at the TIVAP, the most frequently diagnosed diseases were gastrointestinal tumors, followed by breast tumors. There was no difference in age, gender, educational level, body mass index, implantation time, and disease diagnosis between the two groups (*P*>0.05), as shown in Table 1.

**Table 1:** Comparison of general information between the two groups

Item	Intervention group (*n*=42)	Control group (*n*=42)	Statistical value	*P*
Age (years, $\overline{x}$±s)	57.31±12.17	56.05±13.86	0.443	0.659
Gender				
Male	15 (35.7)	23 (54.8)	3.076	0.079
Female	27 (64.3)	19 (45.2)		
Educational level			0.857	0.651
Primary school and below	13 (31.0)	15 (35.7)		
Junior and senior high school	13 (31.0)	15 (35.7)		
College and above	16 (38.1)	12 (28.6)		
Body mass index (kg/m^2^,$\overline{x}$±s)	21.72±3.90	22.37±3.90	−0.770	0.443
Implantation time [months, M (QR) ]	15(14,16)	15(14,16)	−0.224	0.823
Diagnosis			7.091	0.069
Breast tumor	16 (38.1)	15 (35.7)		
Gastrointestinal tumor	16 (38.1)	22 (52.4)		
Respiratory tract tumor	4 (9.5)	5 (11.9)		
Gynecological tumor	6 (14.3)	0 (0.0)		

### Main outcome measures

Table 2 shows the main outcome indicators for both groups of patients. The pain score in the intervention group was less than half that of the control group, with 50% patients in the control group experiencing moderate to severe pain, while only 2 (4.8%) patients in the intervention group experiencing moderate pain. The success puncture rate of the first time puncture in both groups was 100%. The median score of comfortability in the two groups was 10, but 10 patients in the control group reported a comfortability score of less than 10, and 3 patients in the intervention group reported a comfortability score of less than 10, so the comfortability score of the intervention group tilted to the right, leading to differences between groups. In terms of acceptance, 78.6% of the patients in the intervention group said they would continue to choose this spray in the future, while only 28.6% of the patients in the control group would continue to choose this spray in the future. The acceptance of spray in the control group was far lower than that in the intervention group. One week after needle maintenance, 84 patients in the control group and the intervention group were followed up, and only one patient complained of mild itching at the puncture site, which spontaneously relieved the next day. No other adverse reactions occurred.

**Table 2:** Outcome measures in patients undergoing puncture according to allocation to intervention (lidocaine spray) or control (water spray)

	Intervention group (*n*=42)	Control group (*n*=42)	Statistical value	*P*-value
Pain with puncture (mm, $\overline{x}$±s)	15.12±6.61	36.50±18.79	−6.957	< 0.001
Degree of pain			22.558	< 0.001
No pain	5 (11.9)	1 (2.4)		
Mild pain	35 (83.3)	20 (47.6)		
Moderate pain	2 (4.8)	18 (42.9)		
Severe pain	0 (0)	3 (7.1)		
Successful puncture rate of the first time puncture	42 (100)	42 (100)		
Comfort with spray [score, M (QR)]	10 (10–10)	10 (9.75–10)	−2.190	0.028
Patient willing to have the same spray in the future			21.210	< 0.001
Yes	33 (78.6)	12 (28.6)		
Not sure	7 (16.7)	25 (59.5)		
No	2 (4.8)	4 (9.5)		
Adverse reactions at follow-up	1 (2.4)	0 (0)	0.000	1.00

## Discussion

To our knowledge, this is the first study on the analgesic effect of lidocaine spray on non-coring needle puncture. We found that lidocaine spray was effective in relieving pain due to TIVAP puncture in patients, with high patient acceptance and low incidence of adverse reactions. In addition, although the median comfortability score of patients in the intervention group was the same as that of the control group, but there was a significant difference in comparison between the two groups; the successful puncture rate of the first time puncture was 100% as in the control group.

Compared to conventionally used injection needles, non-coring needles contain a folding point and have a slightly longer bevel with a smaller angle, which puncture at a 90-degree angle to the injection seat with only a portion of the force acting on the needle tip, making the needle slower to pass through the skin and easily causing pain to the patient. Our results showed that the pain score of patients in the lidocaine spray intervention group was 21.38 mm lower than that in the control group, and the proportion of patients with moderate and severe pain was significantly lower than that of the control group, indicating that lidocaine spray can effectively reduce the pain caused by the non-coring needle puncture of patients implanted with TIVAP. Our results were inconsistent with the findings of Joris Datema [22] that the local administration of lidocaine spray failed to effectively relieve the pain of adult patients during intravenous intubation. However, İsmail Ufuk Yıldız [19] also indicated that lidocaine spray contributed to relieving the pain of adult patients during radial artery puncture, which was consistent with the results of this study. The underlying reasons may be individual differences, different doses of lidocaine spray, and different puncture sites. Lidocaine spray is an amide anesthetic that can penetrate the skin. Its main component lidocaine can stabilize the nerve membrane by inhibiting the ion flow required for generating and conducting exciting waves, thus producing local anesthesia.

Notably, 23.8% of the patients in the intervention group still felt mild pain during non-coring needle puncture, indicating that the lidocaine spray could not completely eliminate the pain caused by the non-coring needle puncture of the patients with TIVAP. Previous studies pointed out that lidocaine spray was not enough to eliminate the pain related to venous puncture [25]. These results may be attributed to individual differences, different doses of lidocaine spray, and different puncture sites. In the future, we can further explore the exact optimal dose of lidocaine spray to control the pain caused by needle insertion.

Although the median score of comfortability for both groups of patients was 10, the difference in the comfortability score between the two groups was statistically significant. The local anesthetic effect of lidocaine spray on the skin might reduce the stimulation to the patient’s skin during disinfection and alleviate the pain of non-coring needle puncture, thus relieving anxiety and improving the comfortability of the patient. In terms of acceptance, 78.6% of patients in the intervention group reported that they were willing to choose this spray to reduce intubation pain in the future, while the acceptance in the control group was only about 28.6%, reflecting the patient satisfaction with lidocaine spray. Firstly, patients can directly benefit from lidocaine spray, that is, reducing the pain during needle puncture. Secondly, lidocaine spray can produce a mellow aroma when sprayed out. In addition, one significant advantage of lidocaine spray in pain management related to non-coring needle puncture is that it can reduce the pain of non-coring needle puncture and has no need for injection. Moreover, the effect of alleviating pain can be achieved within 5 min after the application of lidocaine spray, while local anesthetic cream and gel usually require a waiting period of at least 30 min. Hence, the use of the spray can save a lot of waiting time for patients and medical staff.

In this study, only one patient in the intervention group complained of slight itching on the skin at the puncture site after the non-coring needle puncture, but it was quickly relieved. There were no other adverse reactions during the 1 week of follow-up. Many factors lead to skin itching, and it is difficult to determine whether the itching is caused by lidocaine spray. It is reported that 27.6% of patients have white skin 5 min after non-coring needle puncture with lidocaine cream for local anesthesia [8]. Compared with lidocaine cream, lidocaine spray means a lower incidence of adverse reactions, and there is no statistically significant difference between the two groups of patients.

## Limitation of this study

This study is a randomized controlled trial that can effectively control bias and ensure study quality. However, this study is a single-center study with insufficient sample representation. To enhance the reliability of lidocaine spray in reducing needle puncture pain in cancer patients implanted with TIVAP, a multi-center study with a larger sample size is required. Moreover, this study did not assess individual pain sensitivity and pre-maintenance anxiety, which may affect the degree of individual pain. Further intervention research is warranted in the future.

## Conclusion

This study provides evidence that lidocaine spray can reduce pain caused by non-coring needle puncture at the TIVAP, which can be used for pain management related to non-coring needle puncture. However, some patients still have mild pain, and the optimal dose of lidocaine spray for pain control remains further exploration in the future.

## Data Availability

Data will be made available on request.
